# Assessment of Factors Associated With Long-term Posttraumatic Stress Symptoms Among 56 388 First Responders After the 2011 Great East Japan Earthquake

**DOI:** 10.1001/jamanetworkopen.2020.18339

**Published:** 2020-09-29

**Authors:** Masanori Nagamine, Erik J. Giltay, Jun Shigemura, Nic J. van der Wee, Taisuke Yamamoto, Yoshitomo Takahashi, Taku Saito, Masaaki Tanichi, Minori Koga, Hiroyuki Toda, Kunio Shimizu, Aihide Yoshino, Eric Vermetten

**Affiliations:** 1 Division of Behavioral Science, National Defense Medical College Research Institute, Tokorozawa City, Japan; 2 Department of Psychiatry, Leiden University Medical Center, Leiden, the Netherlands; 3 Department of Psychiatry, School of Medicine, National Defense Medical College, Saitama, Japan; 4 ARQ National Psychotrauma Center, Diemen, the Netherlands

## Abstract

**Importance:**

First responders are at risk for developing symptoms of posttraumatic stress disorder (PTSD). Little is known about the risk factors for developing PTSD during a years-long period after complex mass disasters.

**Objective:**

To explore the long-term course of PTSD symptoms and to identify risk factors and their relative association with PTSD among first responders dispatched to the 2011 Japanese earthquake, tsunami, and nuclear disaster.

**Design, Setting, and Participants:**

This 6-year, large, prospective cohort study was part of a continuous longitudinal study of Japan Ground Self-Defense Force first responders. The data were collected at 1, 6, 12, 24, 36, 48, 60, and 72 months after mission completion from 2011 to 2017. Of approximately 70 000 eligible participants, 56 388 were enrolled in this study. Data were analyzed from 2017 to 2020.

**Exposures:**

Stress exposures owing to personal or professional disaster experience (eg, duties with body recovery or radiation exposure risk) and working conditions (eg, deployment length, postdeployment overtime work).

**Main Outcomes and Measures:**

The Impact of Event Scale–Revised score assessed PTSD symptoms; scores of at least 25 were defined as probable PTSD. Cox proportional hazards regression models assessed the risk factors for incidence of probable PTSD.

**Results:**

Among the 56 388 participants, 97.1% were men, and the median age at enrollment was 34 (range, 18-63) years. A probable PTSD rate was 2.7% at 1 month and showed a downward trend in the first year and a subsequent plateau. The cumulative incidence of probable PTSD was 6.75%. The severity of PTSD symptoms demonstrated a high degree of rank-order stability over time. Rather than professional disaster experience, sociodemographic factors and working conditions were independently associated with the incidence of probable PTSD: personal experience of the disaster (hazard ratio [HR], 1.96; 95% CI, 1.72-2.24), deployment length of at least 3 months (HR vs <1 month, 1.75; 95% CI, 1.52-2.02), increased age (HR for ≥46 vs ≤25 years, 2.28; 95% CI, 1.79-2.92), and postdeployment overtime work of at least 3 months (HR vs little to none, 1.61; 95% CI, 1.39-1.87).

**Conclusions and Relevance:**

Given these findings, in the future, first responders’ PTSD symptoms might be mitigated by shortening deployment length, avoiding postdeployment overtime work, and paying special attention to the needs of personnel with personal experience of the disaster or older age. Efforts to alleviate responders’ initial symptoms will be required.

## Key Points

**Question:**

What are the risk factors associated with developing posttraumatic stress disorder (PTSD) among first responders deployed to the 2011 Japanese earthquake, tsunami, and nuclear disaster?

**Findings:**

In this 6-year cohort study of 56 388 first responders, a strong association was found between PTSD and sociodemographic factors (ie, personal experience of the disaster, increased age) and working conditions (ie, deployment length, postdeployment overtime work).

**Meaning:**

These findings suggest that symptoms of PTSD among first responders in mass disasters may be mitigated by providing accommodation or additional support to personnel with personal experience of the disaster or increased age as well as monitoring deployment length and postdeployment overtime work.

## Teaser

This cohort study explores the long-term course of posttraumatic stress disorder (PTSD) symptoms and identifies risk factors and their relative association with posttraumatic stress disorder among first responders dispatched to the 2011 Japanese earthquake, tsunami, and nuclear disaster.

## Introduction

On March 11, 2011, a magnitude 9.0 giant earthquake struck Japan and subsequently caused tsunamis and a level 7 critical nuclear accident according to the International Nuclear and Radiological Event Scale.^1^ These series of events caused by the Great East Japan Earthquake (GEJE) resulted in 15 897 fatalities, 2533 missing persons, and more than 400 000 buildings destroyed.^2^ The Japanese government immediately dispatched approximately 107 000 Japan Ground Self-Defense Forces (JGSDF) first responders to the affected areas. Disaster relief missions included rescue duties, body recovery operations of approximately 10 000 human remains, and humanitarian support in the areas with a risk of radiation exposure.^3^

First responders involved in disaster relief fulfill their duties amid life-threatening and potentially traumatic situations. They are often also exposed to overwhelming emotional reactions from those affected by the disaster and their families, which could lead to secondary traumatic stress, compassion fatigue,^4^ and vicarious traumatization.^5^ As a result, first responders could manifest posttraumatic stress symptoms, and some responders will develop stress-related disorders, such as posttraumatic stress disorder (PTSD).^6^ A meta-analysis of studies on first responders^7^ estimated the incidence of full-blown PTSD among them at approximately 10%. Major risk factors for PTSD in first responders usually relate to duty, such as earlier start date or longer duration of time working at a disaster site,^8,9,10,11^ exposure to human remains,^12,13,14^ or nuclear disaster response.^15,16^ Other risk factors include female sex,^10,17,18^ direct personal experience of the disaster,^8,9,10,19^ low social support,^17,18,19^ and postdisaster life stressors, such as job loss.^8,9,17,18,19^

Studies to date show that PTSD symptoms tend to develop in complex ways after the event, the degree of which varies depending on the type of event, population, and its time course.^20,21^ Approximately one-fourth of clinical cases are of the delayed-onset type (ie, full-blown PTSD≥6 months after the event).^22^ Long-term longitudinal studies are essential to better understand the development of PTSD symptoms among first responders. Nonetheless, most first responder studies are cross-sectional,^23,24^ and large-scale, long-term longitudinal studies are available primarily from the September 11, 2001, terrorist attacks^9,13,18^ but not from large-scale natural disasters complicated by a nuclear event.

In the case of GEJE first responders, their disaster exposure was unprecedented and complex. In an earlier study, investigators reported not only the psychological effect of the GEJE on JGSDF first responders in the first year but also the risk factors for elevated PTSD symptoms in this time period.^10^ However, long-term assessment of PTSD symptoms in this population was still missing. To fill this gap, we conducted a 6-year longitudinal cohort study on JGSDF first responders. Our aims were to explore the course of PTSD symptoms and to identify the risk factors and their independent association with incident PTSD.

## Methods

### Study Design

This cohort study presents the 6-year follow-up, a continuation of an initial 1-year longitudinal study on JGSDF first responders dispatched to the GEJE.^10^ In the initial study, self-report questionnaires were sent to approximately 70 000 JGSDF personnel, and the authors investigated PTSD symptoms at 1, 6, and 12 months after mission completion. For the present study, the database of the initial study was combined with the data of PTSD symptoms obtained from the annual health surveys from JGSDF personnel from 2013 to 2017 (ie, 2 to 6 years after mission completion). Of the 56 753 participants in the initial study, we excluded from the data those participants whose organizational service numbers were missing. Finally, 56 388 JGSDF personnel deployed for the GEJE were enrolled in this study (eFigure 1 in the Supplement). We double-checked the validity of the merged databases by including the key question item “Have you ever been dispatched to the GEJE disaster relief mission?” in the annual health survey.

Because this study was conducted as part of an occupational health program in the JGSDF, written informed consent was not obtained from each participant. Instead, we disclosed the study objectives and procedure (all data were anonymized before the analyses) to the participants and provided them with the opportunity to refuse participation. All procedures in this study complied with the ethical standards of the relevant national and institutional committees on human experimentation and with the Declaration of Helsinki of 1975, as revised in 2013.^25^ Approval to perform this research was obtained from the Ethics Committee of the National Defense Medical College, Tokorozawa, Japan. This study follows the Strengthening the Reporting of Observational Studies in Epidemiology (STROBE) reporting guideline.

### Exposure

Data for this study were collected from 2011 to 2017. At the first-year assessment, information on sociodemographic factors (ie, sex, age, rank, and personal experience of the disaster), professional disaster experience (ie, body recovery, duties with radiation exposure risk), and working conditions (ie, deployment length, postdeployment leave, and postdeployment overtime work) were collected. Plain, dichotomous questions were used to assess whether respondents were personally affected by the disaster (yes or no), whether first responders had performed duties related to body recovery, and to ascertain first responders’ risk of radiation exposure. Information about postdeployment factors (ie, timing of postdeployment leave, extent of postdeployment overtime work) was collected using selective-answer questions on a 12-month postmission survey. Working overtime was defined as working outside duty hours or on holidays.

### Main Outcome Measure

Symptoms of PTSD were evaluated using the Impact of Event Scale–Revised (IES-R) score.^26^ An IES-R score of 25 or more was validated as indicating high risk for PTSD in the Japanese sample^27^; we defined this range (25-88) as probable PTSD. The previous study demonstrated good test-retest reliability (*r* = 0.86; *P* < .001) and high internal consistency (Cronbach coefficient α in total score, 0.92-0.95) of this scale.^27^ We collected the IES-R data at as many as 8 points throughout the survey period, including 1, 6, and 12 months after the mission in the initial follow-up study and from 2 to 6 years after the mission annually in the long-term follow-up survey. To check the temporal trends in those participants with probable PTSD, we classified them into 3 groups (*recovered* if they scored <25 on the IES-R; *persisted* if they scored ≥25 on the IES-R continuously; and *recurrent* if they scored ≥25 on the IES-R but scored <25 on the most recent IES-R). The classification was performed for those with probable PTSD at baseline (1 month) throughout the survey period.

### Statistical Analysis

Data were analyzed from 2017 to 2020. Data analyses were conducted using the open source R statistical software, version 3.4.0 (R Foundation for Statistical Computing) and RStudio, version 1.1.447 (R Foundation for Statistical Computing). Because the IES-R scores were strongly positively skewed, scores were naturally logarithmically transformed before the analyses. Trajectories of geometric mean values (with 95% CIs) are shown according to categories of baseline IES-R scores. In a sensitivity analysis, these analyses were repeated in the subgroup of participants with 6 or more assessments over time. Reliability coefficients (ie, an intraclass correlation coefficient with a 1-way random-effects model with single-measure reliability) were used to examine temporal stability (using the icc function in R).

The Kaplan-Meier method was used to present crude incidences of probable PTSD, excluding those participants with IES-R scores of at least 25 at baseline (ie, 1 month after the deployment). The proportional hazards assumption was checked using the graphical diagnostics based on the graphs of the log(–log[survival]) vs log of survival time. We checked whether plots showed nonrandom patterns against time (using the cox.zph and cloglog functions and in the survival and survminer packages in R [R Foundation for Statistical Computing]). We found no evidence of violation of the proportional hazards assumption (eFigure 2 in the Supplement). Hazard ratios (HRs) with 95% CIs of probable incidence of PTSD were estimated using univariate and multivariate Cox proportional hazards regression models. Relative associations of each independent variable with incident PTSD were evaluated using *z* scores, the ratio of each regression coefficient to its standard error (SE) (ie, coefficient/SE[coefficient]). In sensitivity analyses, multivariate models were tested separately in strata with low (ie, 0-4) and high (ie, 5-24) IES-R scores at baseline and in strata with and without personal experience of the disaster. A 2-tailed *P* < .05 was considered statistically significant.

## Results

Of the 56 388 participants, 1620 were women (2.9%) and 54 768 were men (97.1%); the median age at enrollment was 34 (range, 18-63) years. The Table demonstrates baseline sociodemographic variables and the IES-R scores of first responders throughout the survey period. Because the number of JGSDF personnel deployed for the GEJE has not been precisely reported but could be estimated at approximately 70 000, the participation rate was estimated at 80.6%. There was a gradual attrition during follow-up over time from 99.3% at 1 year to 44.3% at 6 years (Table). Although the follow-up participation rates decreased over time, 50 980 (90.4%) of the 56 388 participants were followed up at least once during the 2- through 6-year follow-up waves. The comparison of baseline PTSD symptoms between participants with and without follow-up after a 2-year survey period is shown in eTable 1 in the Supplement, indicating that participants who were subject to attrition had more PTSD symptoms than those continuing to participate (mean [SD] IES-R score among participants with and without follow-up after a 2-year survey period, 4.5 [7.2] vs 5.3 [8.1]). This eTable also represents the JGSDF system in which officers and sergeants have permanent employment, whereas privates have a fixed-term employment. Although private rank accounted for just 12.7% of participants followed up after the 2-year survey point, it accounted for 42.3% of those who dropped out during that period. Distribution and the 75th and 90th percentiles of IES-R scores in density plots show that the proportion of participants with more severe PTSD symptoms (area of density plots for IES-R score >25) tended to decline over time (eFigure 3 in the Supplement). The probable PTSD ratio at each survey point decreased over time from 2.7% at 1 month to 1.0% at 6 years (eFigure 4 in the Supplement). The cumulative incidence of probable PTSD was 6.75% throughout the survey period (using the Kaplan-Meier estimate).

**Table.** Baseline Sociodemographic Variables and IES-R Scores Over Time in Participants During Follow-up to 72 Months

Variable	Total No. of participants	Follow-up, mo^a^							
		1	6	12	24	36	48	60	72
Participants followed up	56 388 (100)	53 700 (95.2)	55 155 (97.8)	56 006 (99.3)	41 218 (73.1)	33 894 (60.1)	27 477 (48.7)	29 889 (53.0)	24 999 (44.3)
IES-R score									
Mean (SD)	NA	4.6 (7.3)	3.6 (6.2)	2.9 (5.4)	2.9 (5.8)	2.0 (4.9)	1.9 (4.9)	2.4 (5.9)	2.0 (5.0)
Median (IQR)	NA	2 (0-6)	1 (0-4)	1 (0-3)	1 (0-3)	0 (0-2)	0 (0-1)	0 (0-2)	0 (0-2)
Probable PTSD	3319 (5.9)^b^	1475 (2.7)	941 (1.7)	644 (1.1)	562 (1.4)	315 (0.9)	254 (0.9)	463 (1.5)	252 (1.0)
Sex									
Male	54 768 (97.1)	52 191 (97.2)	53 580 (97.1)	54 405 (97.1)	40 254 (97.7)	33 157 (97.8)	26 882 (97.8)	29 265 (97.9)	24 438 (97.8)
Female	1620 (2.9)	1509 (2.8)	1575 (2.9)	1601 (2.9)	964 (2.3)	737 (2.2)	595 (2.2)	624 (2.1)	561 (2.2)
Age, y									
≤25	10 209 (18.1)	9648 (18.0)	9874 (17.9)	10 084 (18.0)	6235 (15.1)	4919 (14.5)	3865 (14.1)	4286 (14.4)	3455 (13.8)
26-30	10 559 (18.7)	10 086 (18.8)	10 302 (18.7)	10 486 (18.7)	7847 (19.1)	6847 (20.2)	5665 (20.6)	6228 (20.9)	5226 (20.9)
31-35	9637 (17.1)	9205 (17.2)	9430 (17.1)	9600 (17.2)	7672 (18.6)	6533 (19.3)	5572 (20.3)	6164 (20.6)	5370 (21.5)
36-40	8231 (14.6)	7852 (14.6)	8089 (14.7)	8192 (14.6)	6550 (15.9)	5549 (16.4)	4630 (16.9)	5166 (17.3)	4563 (18.3)
41-45	7873 (14.0)	7512 (14.0)	7741 (14.0)	7831 (14.0)	6337 (15.4)	5327 (15.7)	4456 (16.2)	4953 (16.6)	4315 (17.3)
≥46	9831 (17.4)	9353 (17.4)	9673 (17.6)	9765 (17.5)	6540 (15.9)	4692 (13.9)	3269 (11.9)	3070 (10.3)	2048 (8.2)
Rank									
Officer	6398 (11.3)	5901 (11.0)	6176 (11.2)	6324 (11.3)	4566 (11.1)	3390 (10.0)	2629 (9.6)	3065 (10.3)	2867 (11.5)
Sergeant	41 205 (73.1)	39 455 (73.5)	40 420 (73.3)	41 032 (73.3)	31 827 (77.2)	26 847 (79.2)	21 974 (80.0)	23 662 (79.2)	19 594 (78.4)
Private	8785 (15.6)	8344 (15.5)	8559 (15.5)	8650 (15.4)	4825 (11.7)	3657 (10.8)	2874 (10.5)	3162 (10.6)	2538 (10.2)
Deployment length, mo									
<1	23 609 (41.9)	22 168 (41.3)	23 085 (41.9)	23 440 (41.9)	16 904 (41.0)	13 361 (39.4)	10 476 (38.1)	11 901 (39.8)	10 245 (41.0)
1-2	25 002 (44.4)	24 206 (45.1)	24 466 (44.4)	24 854 (44.4)	18 643 (45.2)	15 703 (46.3)	13 114 (47.7)	14 062 (47.1)	11 993 (48.0)
≥3	7746 (13.7)	7311 (13.6)	7574 (13.7)	7681 (13.7)	5660 (13.7)	4822 (14.2)	3880 (14.1)	3916 (13.1)	2757 (11.0)
Timing of postdeployment leave									
Early	32 229 (57.7)	30 909 (58.1)	31 584 (57.8)	32 220 (57.7)	23 930 (58.5)	19 758 (58.8)	16 872 (61.9)	17 679 (59.6)	15 232 (61.4)
Late	20 157 (36.1)	19 059 (35.8)	19 680 (36.0)	20 150 (36.1)	14 521 (35.5)	11 863 (35.3)	8942 (32.8)	10 317 (34.8)	8328 (33.6)
None	3465 (6.2)	3216 (6.0)	3356 (6.1)	3464 (6.2)	2441 (6.0)	2001 (6.0)	1427 (5.2)	1656 (5.6)	1258 (5.1)
Postdeployment overtime work									
Little to none	37 363 (66.9)	35 615 (67.0)	36 523 (66.9)	37 351 (66.9)	26 958 (66.0)	22 419 (66.7)	17 942 (65.9)	19 556 (66.0)	16 240 (65.5)
<3 mo	13 320 (23.9)	12 678 (23.9)	13 043 (23.9)	13 315 (23.9)	9998 (24.5)	8082 (24.1)	6655 (24.4)	7176 (24.2)	6077 (24.5)
≥3 mo	5136 (9.2)	4861 (9.1)	5022 (9.2)	5135 (9.2)	3905 (9.6)	3091 (9.2)	2627 (9.6)	2903 (9.8)	2483 (10.0)
Personal experience of the disaster									
No	51 356 (91.2)	48 949 (91.2)	50 257 (91.2)	51 014 (91.2)	37 509 (91.1)	30 858 (91.1)	24 957 (90.9)	27 359 (91.6)	23 089 (92.5)
Yes	4983 (8.8)	4704 (8.8)	4851 (8.8)	4944 (8.8)	3668 (8.9)	3005 (8.9)	2488 (9.1)	2496 (8.4)	1884 (7.5)
Body recovery duties									
No	39 635 (70.3)	37 553 (70.0)	38 856 (70.5)	39 351 (70.3)	28 919 (70.2)	23 158 (68.4)	18 558 (67.6)	20 355 (68.1)	17 626 (70.5)
Yes	16 734 (29.7)	16 128 (30.0)	16 280 (29.5)	16 636 (29.7)	12 283 (29.8)	10 723 (31.6)	8907 (32.4)	9523 (31.9)	7364 (29.5)
Duties with radiation exposure risk									
No	47 053 (83.5)	44 875 (83.6)	46 100 (83.6)	46 742 (83.5)	34 400 (83.5)	28 198 (83.2)	22 528 (82.1)	24 688 (82.6)	20 785 (83.2)
Yes	9303 (16.5)	8796 (16.4)	9023 (16.4)	9232 (16.5)	6791 (16.5)	5678 (16.8)	4926 (17.9)	5186 (17.4)	4197 (16.8)

Abbreviations: IES-R, the Impact of Event Scale–Revised; IQR, interquartile range; NA, not applicable; PTSD, posttraumatic stress disorder.

^a^ Unless otherwise indicated, data are expressed as number (percentage) of participants. Percentages have been rounded and may not total 100. Owing to missing data, numbers do not all sum to column heading.

^b^ The value demonstrates cumulative incidence of probable PTSD (scores ≥25 on the IES-R) throughout the survey period.

**Figure 1.**
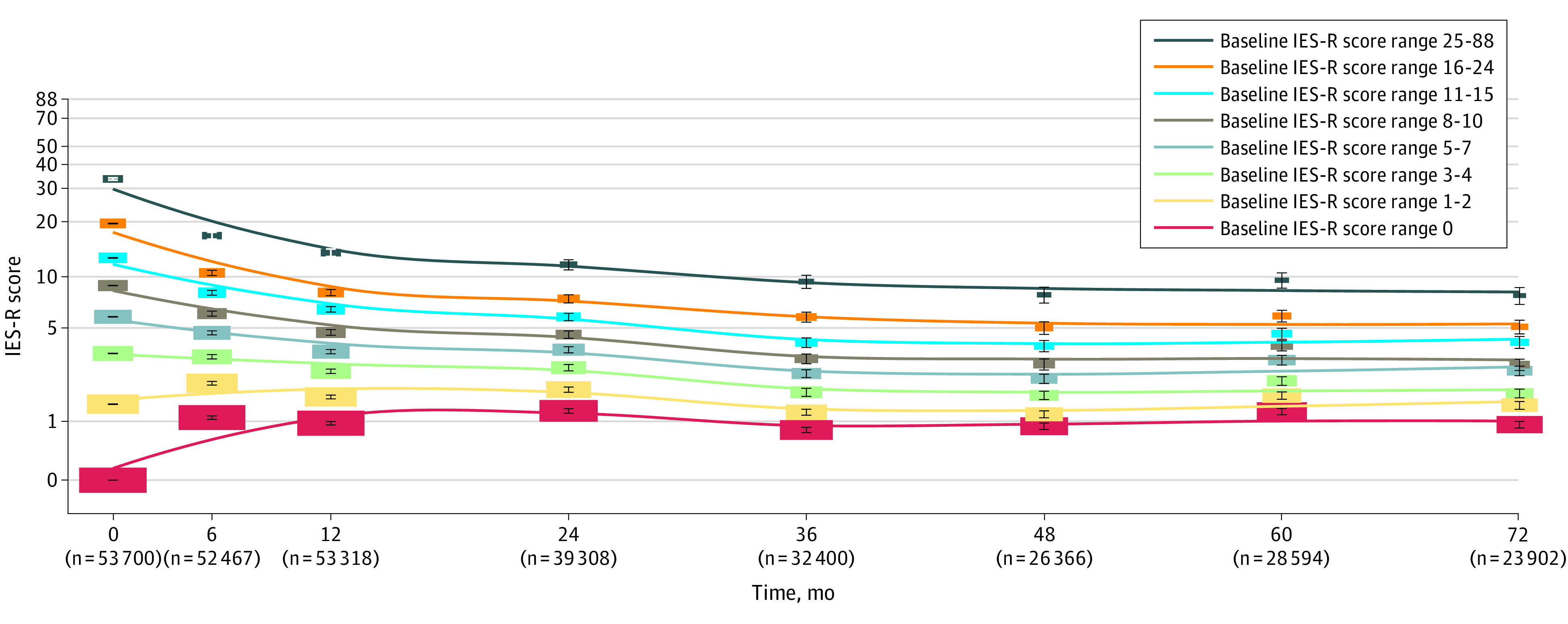
Change in Mean Impact of Event Scale–Revised (IES-R) Scores Over Time by Baseline Score on a Logarithmic Scale Stratified categories are based on baseline IES-R scores. At 1 month, 19 361 participants (36.1%) had scores of 0; 11 279 (21.0%), scores of 1 to 2; 6635 (12.4%), scores of 3 to 4; 5702 (10.6%), scores of 5 to 7; 3483 (6.5%), scores of 8 to 10; 3122 (5.8%), scores of 11 to 15; 2643 (4.9%), scores of 16 to 24; and 1475 (2.7%), scores of 25 to 88. Error bars represent 95% CIs of the mean, and the size of each box is proportional to the number of participants within that category at that point. Although mean levels of the IES-R declined in those with high baseline IES-R scores, there was evidence of substantial rank-order stability during 72 months of follow-up. Thus, relative IES-R scores of individuals over time were stable.

Figure 1 shows, on a logarithmic scale, the geometric mean changes over time within stratified groups based on baseline IES-R scores (ie, 1 month after mission completion). Although there was a declining trend in geometric mean levels of the IES-R score in participants with high baseline IES-R scores, evidence suggested substantial rank-order stability throughout the 6-year follow-up period. The intraclass correlation coefficient (n = 5686 with complete data on all 8 time points) was 0.38 (95% CI, 0.37-0.39; *F*_5685, 39 802_ = 5.91; *P* < .001). The 7 intraclass correlation coefficients for each pair of adjoining measurements ranged from 0.42 to 0.58. In a sensitivity analysis, there were similar findings in the subgroup of 33 190 participants with at least 6 (of 8) assessments during follow-up (eFigure 5 in the Supplement). Most of those with probable PTSD at baseline recovered over time, although the symptoms persisted or recurred in a given number of them (eTable 2 and eFigure 6 in the Supplement).

**Figure 2.**
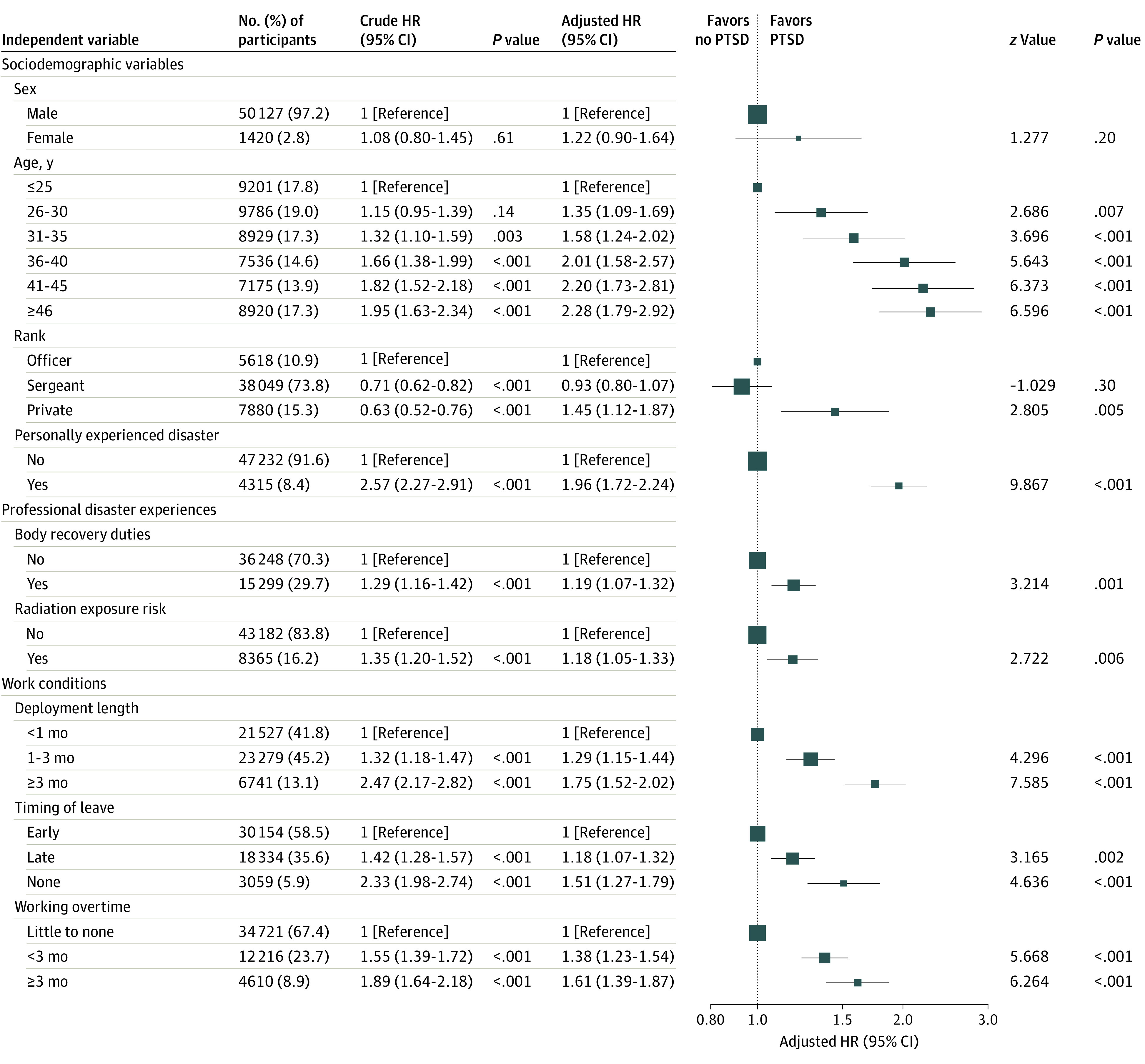
Adjusted Hazard Ratios (HRs) and Test Statistics (*z* Values) of the 9 Baseline Risk Factors for the Incidence of Probable Posttraumatic Stress Disorder (PTSD) Data were analyzed using a multivariate Cox proportional hazards regression model. This model investigated the association between the time to first occurrence of the Impact of Event Scale–Revised (IES-R) score of at least 25 (probable PTSD) and the risk factors. The *z* scores correspond to the ratio of each regression coefficient to its standard error (SE) (ie, coefficient/SE[coefficient]).

The following 4 factors have the strongest association with incidence of probable PTSD in the multivariate Cox proportional hazards regression model for those without probable PTSD during the 1-month survey period (Figure 2): personal experience of the disaster (HR, 1.96; 95% CI, 1.72-2.24; *z* score, 9.87), deployment length of 3 months or more (HR vs <1 mo, 1.75; 95% CI, 1.52-2.02; *z* score, 7.58), older age (HR for ≥46 vs ≤25 years, 2.28; 95% CI, 1.79-2.92; *z* score, 6.60), and postdeployment overtime work of 3 months or more (HR vs little to none, 1.61; 95% CI, 1.39-1.87; *z* score, 6.26). The other baseline variables except for sex were also significantly associated with the incidence of probable PTSD throughout the survey period, both in univariate and fully adjusted models: timing of postdeployment leave (HR for no leave taken vs early leave, 1.51; 95% CI, 1.27-1.79; *z* score, 4.64), body recovery duties (HR, 1.19; 95% CI, 1.07-1.32; *z* score, 3.21), rank (HR for private vs officer, 1.45; 95% CI, 1.12-1.87; *z* score, 2.81), and radiation exposure risk (HR, 1.18; 95% CI, 1.05-1.33; *z* score, 2.72). Kaplan-Meier curves of probable PTSD also demonstrated that the disparity of curves and χ^2^ statistics was greater, particularly in these influential independent variables (eFigure 7 in the Supplement). In sensitivity analyses related to baseline IES-R scores, these 4 factors with the strongest hazard were associated with similar strengths within both strata with low (ie, 0-4) and high (ie, 5-24) IES-R scores at baseline (eFigures 8 and 9 in the Supplement), which suggests that the findings on these factors were largely independent of baseline IES-R severity. In another sensitivity analysis related to personal experience of the disaster (eFigures 10 and 11 in the Supplement), we found largely similar trends in the results within both strata; however, some factors, including professional disaster experiences, lost significance within a stratum with personal experience of the disaster, supporting the association of personal disaster experience with probable PTSD.

## Discussion

In this multiyear cohort study involving more than 50 000 uniformed GEJE disaster workers, 4 unique and independent risk factors for the probable development of PTSD were identified: personal experience of the disaster, deployment length, age, and postdeployment overtime work. Contrary to our expectation, professional disaster experience (from body recovery or possible radiation exposure missions) was only marginally (although statistically significantly) associated with probable PTSD. The correlates of probable PTSD are largely similar to those found in the initial 1-year longitudinal study,^10^ suggesting that these vulnerability factors also have long-term association with PTSD. These data are of great importance because they clearly indicate opportunities for intervention in future disaster relief efforts.

We found a high degree of stability of the severity of PTSD symptoms during the 6 years of our study.^28^ This stability was shown in the time course analysis of mean PTSD symptoms, stratified according to the baseline PTSD symptoms. Although probable PTSD remitted spontaneously in many first responders within 6 and 12 months, the symptoms persisted, recurred, or intensified in a substantial number of them, possibly in response to reminders of the original trauma. Previous research has also reported a high correlation between acute and late PTSD.^29,30^ Our findings indicate that the trajectory of PTSD symptoms is in part associated with the PTSD symptoms at the initial phase. Increased efforts and interventions to mitigate or even prevent the initial PTSD symptoms might therefore have the potential to lower long-term PTSD symptoms among first responders and should be further investigated or implemented.

In our study, the cumulative incidence (6.75%) and observed point prevalence (2.7% at 1 month to 1.0% at 6 years) of probable PTSD were lower than those in other studies. Prevalence of PTSD among first responders varies depending on the nature and severity of the disaster, the types of responders, and the survey method (eg, survey period, evaluation method). One systematic review^7^ reported that the pooled PTSD prevalence was 10%, ranging from 0 to 46%. At 14 months after the GEJE, a survey of disaster workers^31^ reported that the prevalence of probable PTSD was 6.6% among municipality workers, 6.6% among medical workers, and 1.6% among firefighters. Given the variety of study designs that used different assessment tools, results must be compared cautiously. Nonetheless, possible explanations for lower probable PTSD rates may include recognition of the responders’ efforts by those affected by the disaster and the society,^32,33,34^ fatigue recovery measures used by the JGSDF during the deployment,^35^ and the effectiveness of the long-term mental health follow-up programs, which formed the basis of our investigation.^10^ We should consider the possibility that the Japanese sociocultural background, with a strong stigma against the expression of psychological distress,^36^ might have induced underreporting. Unfortunately, we cannot explore these hypotheses in our data.

Our results confirmed the association between deployment length and PTSD. This trend is compatible with September 11 first responder studies, which report that an earlier start date or longer duration of time worked at a disaster site were associated with greater risk of PTSD.^8,9,11,37^ Our study also identified 2 unique risk factors related to working condition: postdeployment overtime work and failure to take postdeployment leave (or late timing of postdeployment leave). In general, first responders are unexpectedly assigned to their mission, and their routine work accumulates when the mission is completed. This situation often forces first responders to work overtime or to give up taking postdeployment leave. In the military, postdeployment readjustment (from a specific condition to daily life) is considered an important factor to maintain mental health.^38^ Some military organizations provide deployed soldiers with enough rest and psychoeducation before their homecoming to foster better readjustment,^39^ which is consistent with our results. Therefore, our findings imply that PTSD among first responders after disaster deployment could potentially be mitigated by shortening deployment length, providing first responders with enough leave from work just after mission completion, and avoiding a long duration of postdeployment overtime work.

Among the 4 sociodemographic factors in this study, it became clear that personal experience of the disaster was the strongest risk factor for developing probable PTSD (it had the highest *z* statistic [9.87]). In large-scale disasters such as the GEJE, local first responders are also directly affected by the disaster. Their dual roles (responder and participant) can conflict and form a severe psychological burden, inducing a sense of guilt or shame, also known as moral injury.^40^ A meta-analysis on the data from the September 11 attacks also confirmed that being a first responder as well as directly experiencing the disaster was a high-risk factor for developing PTSD.^41^ Therefore, leaders or managers must understand the distress of first responders who also experience the disaster and should develop strategies to support them, helping them to engage in active coping strategies.^42^

We also found that increased age was a risk factor for probable PTSD. The association between age and PTSD has been inconsistent between studies. A study on police responders to the September 11 attacks reported a significant association between increased age and PTSD prevalence.^17^ Other studies, however, reported that being younger is a risk factor for PTSD^7,12,43^; participants in these studies had different ethnic backgrounds. A history of trauma ^44,45^ or disaster experience^46^ may be associated with increased PTSD prevalence. These factors could explain our finding that older adults are at strong risk of developing probable PTSD.

Although previous literature reported substantial stress-related symptoms after body recovery duties^6,47^ or among those with radiation exposure risk,^15^ our study showed a relatively small but significant association regarding participants’ probable PTSD. High morale of first responders or social recognition for their activities might selectively moderate the correlation with their professional disaster experience.^33,34,48^ Regarding body recovery, anticipated stress was reported in a series of soldier studies.^49,50^ The personal effects could be associated with psychological distress via an emotional link between the remains and the disaster workers.^47,51^ The psychological effects of the risk of radiation exposure have the potential to be varied with their risk perception.^52,53,54^ We did not assess these potential confounders, which might explain the attenuated associations of body recovery duties and risk of radiation exposure with probable PTSD.

Female sex was not identified as a risk factor for probable PTSD in this study. Contrary to the previous literature on PTSD in the general population,^55^ a meta-analysis on the first responders also reported a negative association between sex and PTSD.^7^ Some cultural backgrounds shared among first responders (eg, military, police, or firefighter) might contribute to the negative result. Otherwise, the small percentage of women in our study sample (2.9%) limited the statistical power to detect a significant effect of sex in our multivariate analysis.

### Limitations

This study has some limitations. First, we were unable to control for factors such as detailed disaster experience, marital status, medical history, previous disaster experience or psychological trauma, social support, or life stressors occurring after the mission. Second, because we collected data from an occupational health survey rather than from an anonymous survey, the participants may have underreported their symptoms in this study.^56^ Third, participants with severe psychological conditions may have already been retired or unwilling or unable to respond to the health survey (eTable 1 in the Supplement), which could have led us to underestimate probable PTSD. Fourth, owing to the long follow-up period, there was substantial attrition during the study (44.3% participation at 6 years), which may have introduced bias. Finally, we only have IES-R total scores available for the first-year data sets, which makes analysis of the particular PTSD symptoms in more detail impossible.

## Conclusions

In this unique, large-scale, and long-term cohort study on first responders dispatched to the GEJE, we found that severity of PTSD symptoms at the initial phase demonstrated a high degree of rank-order stability during the course of 6 years. As for the risk factors for probable PTSD, personal experience of the disaster, longer deployment length, older age, and postdeployment overtime work were identified as strong independent factors. Resilience in coping with large-scale disasters is an essential part of national security. Thus, it is vital to sustain mental health among first responders before, during, and after disaster exposure so that they may effectively respond to disasters. In future disaster relief work, shortening deployment length, preventing overtime work after mission completion, and offering additional support or accommodation to older personnel, especially those personally affected by the disaster, all have the potential to mitigate long-term adverse psychological effects among first responders. It is important that policy makers take these factors into consideration and develop labor management and mental health strategies for future disasters.

## Notes

Supplement. **eFigure 1.** Flowchart of Study Participants **eFigure 2.** Log-log Plots of the Survival Function **eFigure 3.** Distribution of IES-R Scores in Density Plots **eFigure 4.** Probable PTSD Prevalence and Cumulative Incidence Over Time **eFigure 5.** Mean IES-R Scores Over Time in Participants With 6 or More Assessments **eFigure 6.** Distribution of IES-R Scores in Density Plots for Those With Baseline IES-R ≥25 **eFigure 7.** Kaplan-Meier Curves of Probable PTSD According to 9 Baseline Variables **eFigure 8.** Sensitivity Analysis in Stratum With Baseline IES-R Scores ≤4 **eFigure 9.** Sensitivity Analysis in Stratum With Baseline IES-R Scores >4 **eFigure 10.** Sensitivity Analysis in Stratum of Those Not Being Personally Affected by the Disaster **eFigure 11.** Sensitivity Analysis in Stratum of Those Being Personally Affected by the Disaster **eTable 1.** Comparison of the Initial Survey Data for Participants With and Without Follow-up After the 2-Year Survey Point **eTable 2.** Trends in the Type of Probable PTSD at Baseline (n = 1475) Classified by the Course of Their PTSD Symptoms
