# Supplementary material for: Assessment of Factors Associated With Long-term Posttraumatic Stress Symptoms Among 56 388 First Responders After the 2011 Great East Japan Earthquake
Source: JAMA Netw Open. 2020 Sep 29;3(9):e2018339. doi: 10.1001/jamanetworkopen.2020.18339 (PMC7525349; doi:10.1001/jamanetworkopen.2020.18339)
Supplement: Supplement. — eFigure 1. Flowchart of Study Participants eFigure 2. Log-log Plots of the Survival Function eFigure 3. Distribution of IES-R Scores in Density Plots eFigure 4. Probable PTSD Prevalence and Cumulative Incidence Over Time eFigure 5. Mean IES-R Scores Over Time in Participants With 6 or More Assessments eFigure 6. Distribution of IES-R Scores in Density Plots for Those With Baseline IES-R ≥25 eFigure 7. Kaplan-Meier Curves of Probable PTSD According to 9 Baseline Variables eFigure 8. Sensitivity Analysis in Stratum With Baseline IES-R Scores ≤4 eFigure 9. Sensitivity Analysis in Stratum With Baseline IES-R Scores >4 eFigure 10. Sensitivity Analysis in Stratum of Those Not Being Personally Affected by the Disaster eFigure 11. Sensitivity Analysis in Stratum of Those Being Personally Affected by the Disaster eTable 1. Comparison of the Initial Survey Data for Participants With and Without Follow-up After the 2-Year Survey Point eTable 2. Trends in the Type of Probable PTSD at Baseline (n = 1475) Classified by the Course of Their PTSD Symptoms [file jamanetwopen-e2018339-s001.pdf]

Supplementary Online Content

Nagamine M, Giltay EJ, Shigemura J, et al. Assessment of factors associated with long-term posttraumatic stress symptoms among 56 388 first responders after the 2011 Great East Japan Earthquake. *JAMA Netw Open*. 2020;3(9):e2018339. doi:10.1001/jamanetworkopen.2020.18339

eFigure 1. Flowchart of Study Participants

eFigure 2. Log-log Plots of the Survival Function

eFigure 3. Distribution of IES-R Scores in Density Plots

eFigure 4. Probable PTSD Prevalence and Cumulative Incidence Over Time

eFigure 5. Mean IES-R Scores Over Time in Participants With 6 or More Assessments

eFigure 6. Distribution of IES-R Scores in Density Plots for Those With Baseline IES-R ≥25

eFigure 7. Kaplan-Meier Curves of Probable PTSD According to 9 Baseline Variables

eFigure 8. Sensitivity Analysis in Stratum With Baseline IES-R Scores ≤4

eFigure 9. Sensitivity Analysis in Stratum With Baseline IES-R Scores >4

eFigure 10. Sensitivity Analysis in Stratum of Those Not Being Personally Affected by the Disaster

eFigure 11. Sensitivity Analysis in Stratum of Those Being Personally Affected by the Disaster

eTable 1. Comparison of the Initial Survey Data for Participants With and Without Follow-up After the 2-Year Survey Point

eTable 2. Trends in the Type of Probable PTSD at Baseline (n = 1475) Classified by the Course of Their PTSD Symptoms

This supplementary material has been provided by the authors to give readers additional information about their work.

©2020 Nagamine M et al. *JAMA Network Open*.

eFigure 1. Flowchart of Study Participants

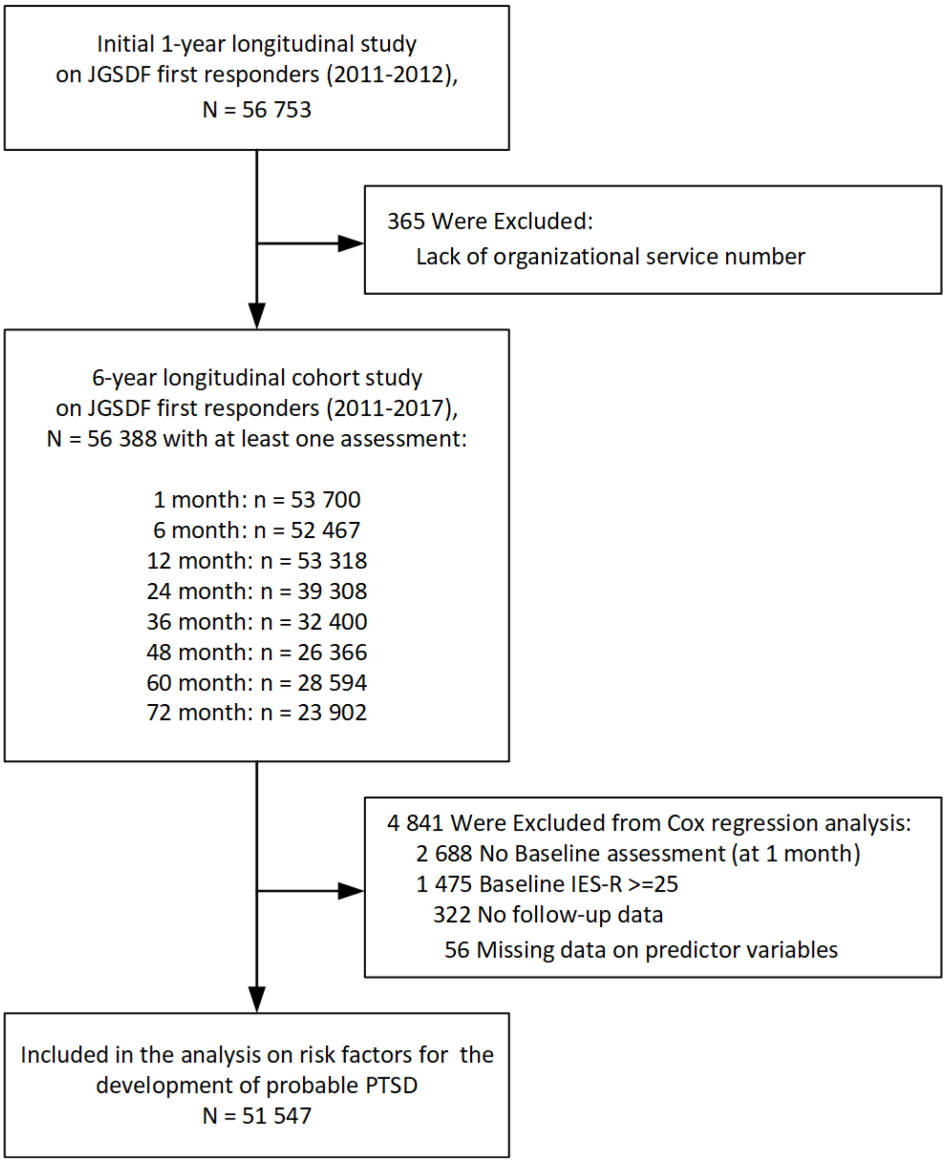

eFigure 2. Log-log Plots of the Survival Function

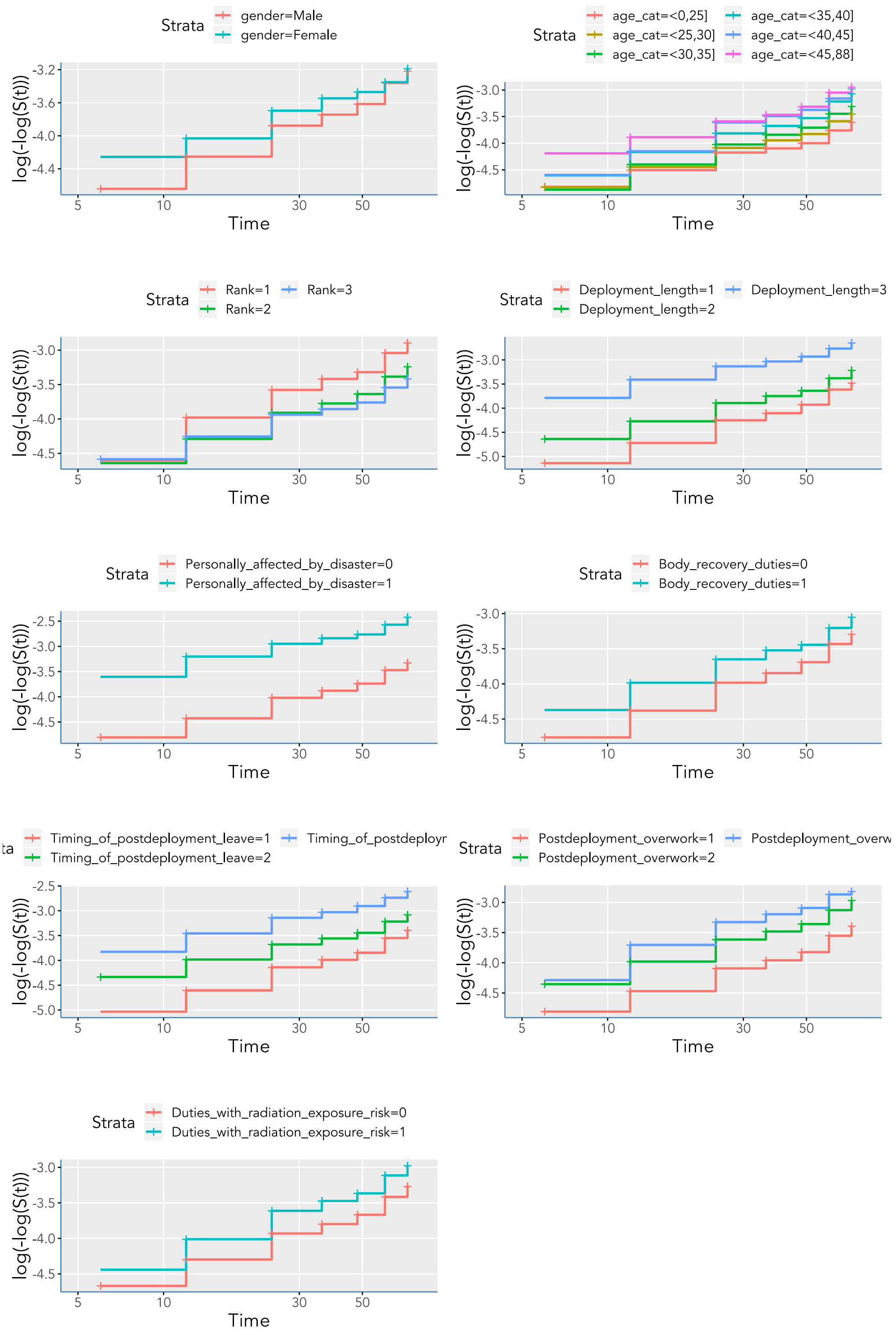

This plot displays the log–log of the survival function, ln(−ln[incident PTSD]), versus the "survival time." A separate plot is shown for each of the 9 baseline variables. These plots provide evidence through the largely parallel (though slightly converging) lines for the idea that the proportional hazards (PH) assumption has not been violated.

©2020 Nagamine M et al. *JAMA Network Open*.

eFigure 3. Distribution of IES-R Scores in Density Plots

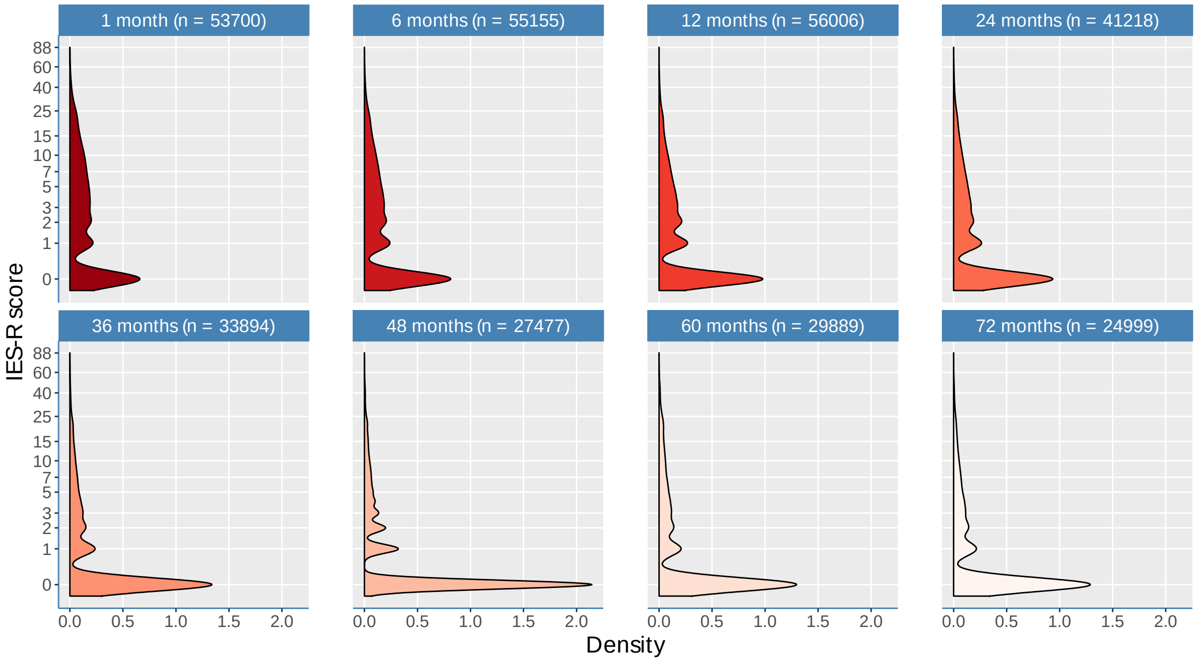

Distribution of IES-R scores in density plots according to the 8 time points during follow up, starting at 1 month after the traumatic event in 53 700 men and women at baseline. The density estimate was scaled to a maximum of 1 (thus showing the relative distributions compared to those with an IESR of 0). The IES-R scores are depicted on a logarithmic scale. Reference lines for probable PTSD are shown.

©2020 Nagamine M et al. *JAMA Network Open*.

eFigure 4. Probable PTSD Prevalence and Cumulative Incidence Over Time

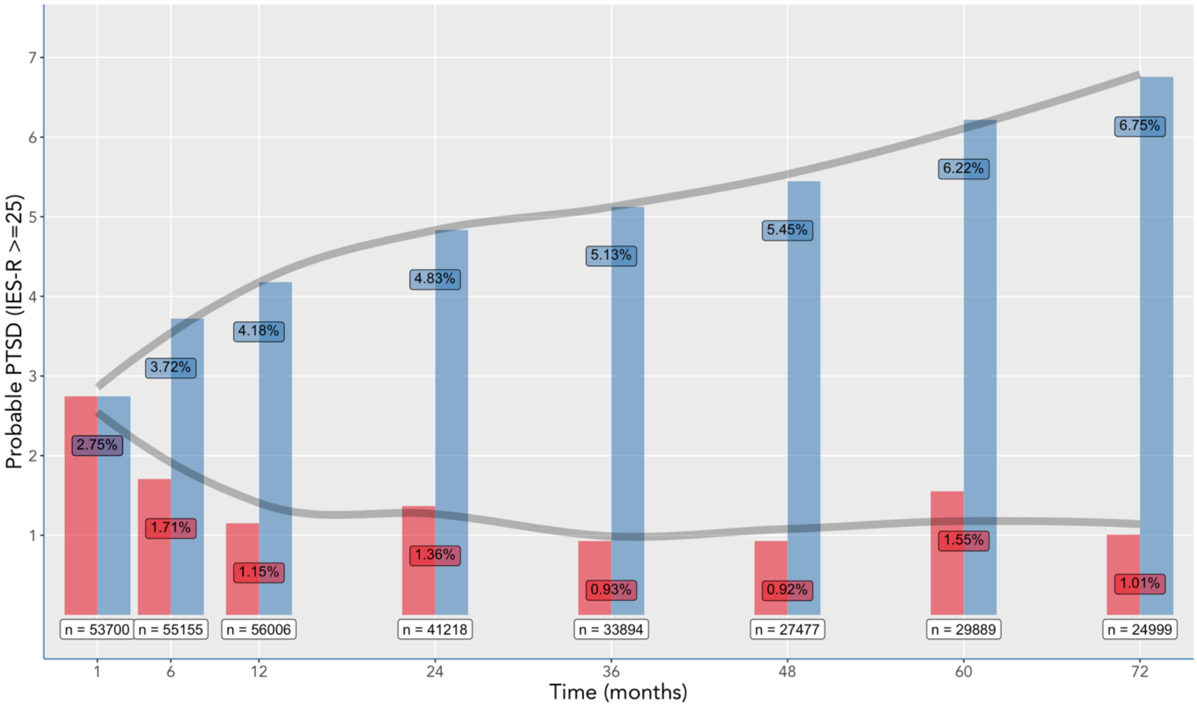

Bar plot with Loess smoothers showing the point prevalence (red bars) and cumulative incidence (blue bars, using the Kaplan–Meier estimate) of participants at each of the 8 time points of follow up with probable PTSD (IES-R score ≥25). The number of participants at each wave is presented above the x axis.

©2020 Nagamine M et al. *JAMA Network Open*.

eFigure 5. Mean IES-R Scores Over Time in Participants With 6 or More Assessments

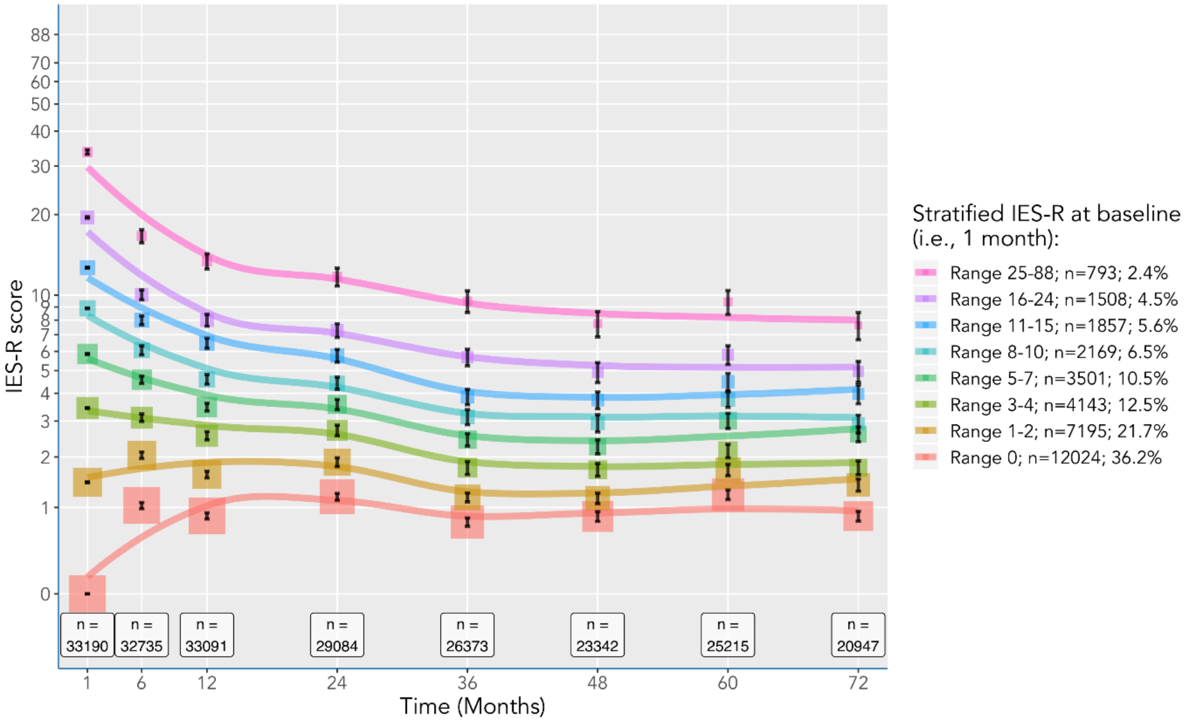

Sensitivity analysis showing the Mean IES-R scores over time in subjects with 6 or more assessments. Error bars represent 95% confidence intervals (CI) of the mean; the size of each box is proportional to the number of subjects within that category at that time point. Less smoother lines are fitted within each of the categories to explore the potential relationships between time and mean IES-R scores.

©2020 Nagamine M et al. *JAMA Network Open*.

eFigure 6. Distribution of IES-R Scores in Density Plots for Those With Baseline IES-R ≥25

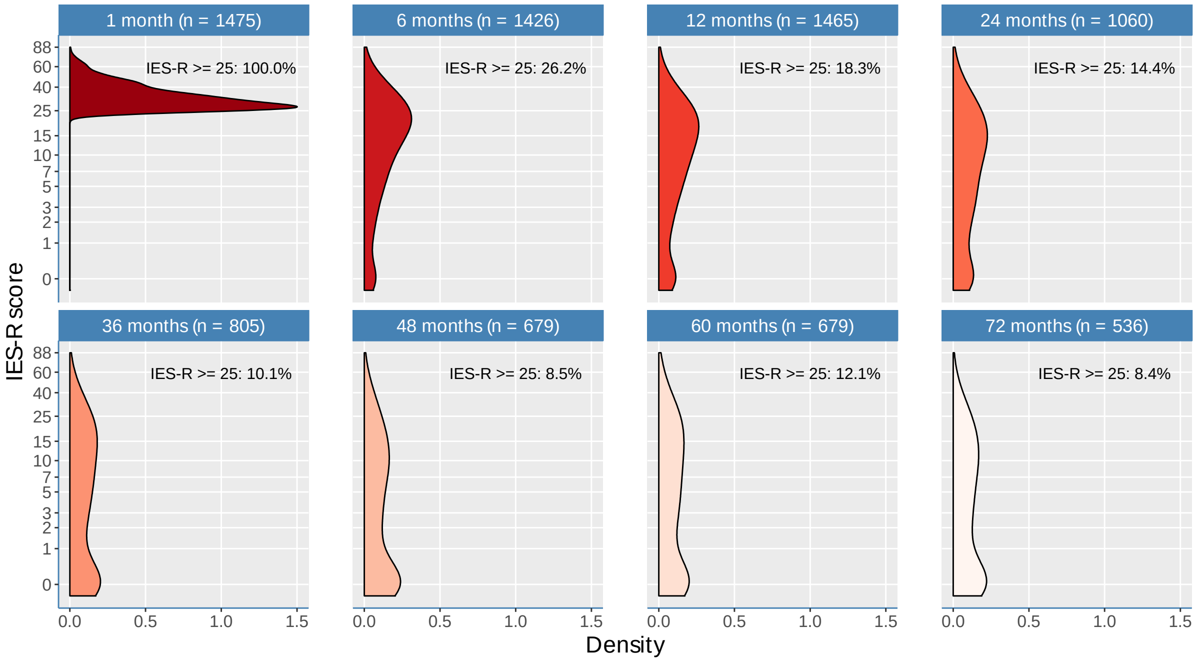

Distribution of IES-R scores in density plots according to the 8 time points during follow up, starting at 1 month after the traumatic event in 1475 men and women at baseline. The density estimate was scaled to a maximum of 1 (thus showing the relative distributions compared to those with an IESR of 0). The IES-R scores are depicted on a logarithmic scale. Reference lines for probable PTSD are shown.

©2020 Nagamine M et al. *JAMA Network Open*.

eFigure 7. Kaplan-Meier Curves of Probable PTSD According to 9 Baseline Variables

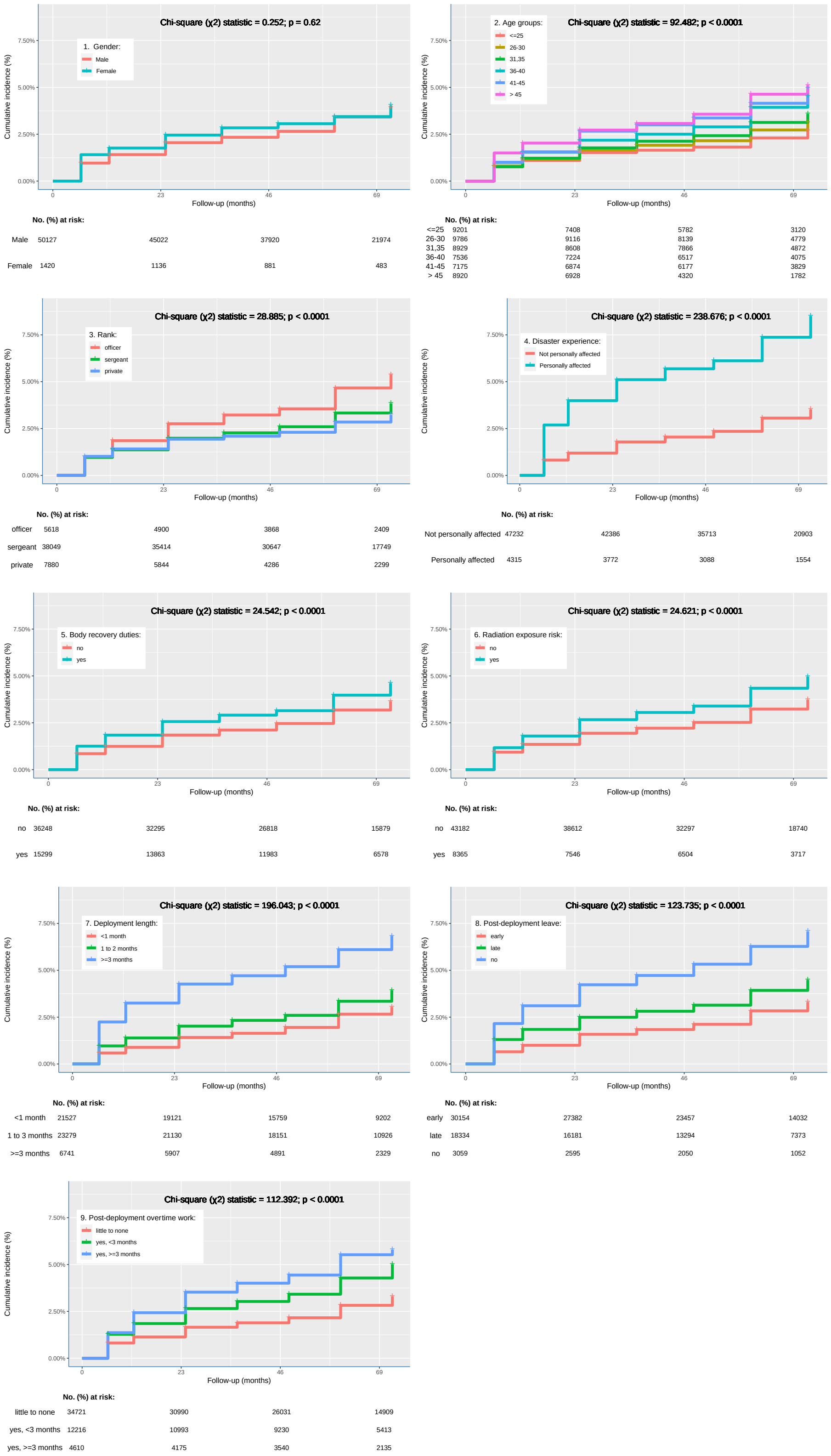

Kaplan–Meier curves of probable PTSD according to 9 baseline predictors for incident probable PTSD. *P* values by log-rank (Mantel–Cox) test.

©2020 Nagamine M et al. *JAMA Network Open*.

eFigure 8. Sensitivity Analysis in Stratum With Baseline IES-R Scores ≤4

Independent variable	n	%	Crude HR	p-value	Adjusted HR	z-value	p-value
**Sociodemographics:**							
1. Gender:							
Male	35832	(97.4%)	1 (ref.)		1 (ref.)		
Female	975	(2.6%)	0.74 (0.41-1.34)	0.32	0.81 (0.44-1.48)	-0.690	0.49
2. Age:							
<= 25 y	6427	(17.5%)	1 (ref.)		1 (ref.)		
26-30 y	7167	(19.5%)	1.22 (0.90-1.65)	0.19	1.38 (0.97-1.96)	1.792	0.07
31-35 y	6597	(17.9%)	1.27 (0.94-1.71)	0.12	1.44 (0.98-2.12)	1.869	0.06
36-40 y	5433	(14.8%)	1.54 (1.14-2.08)	0.005	1.77 (1.20-2.60)	2.888	0.004
41-45 y	5065	(13.8%)	1.79 (1.33-2.41)	< 0.001	2.04 (1.39-3.00)	3.650	< 0.001
>= 46 y	6118	(16.6%)	1.87 (1.38-2.53)	< 0.001	2.09 (1.41-3.09)	3.661	< 0.001
3. Rank:							
Officer	3936	(10.7%)	1 (ref.)		1 (ref.)		
Sergeant	27533	(74.8%)	0.74 (0.59-0.93)	0.01	0.92 (0.72-1.17)	-0.688	0.49
Private	5338	(14.5%)	0.66 (0.48-0.91)	0.01	1.31 (0.86-2.00)	1.276	0.20
4. Disaster experience:							
Not personally affected	34457	(93.6%)	1 (ref.)		1 (ref.)		
Personally affected	2350	(6.4%)	2.07 (1.62-2.63)	< 0.001	1.76 (1.37-2.27)	4.367	< 0.001
**Professional disaster experiences:**							
5. Body recovery duties:							
No	27280	(74.1%)	1 (ref.)		1 (ref.)		
Yes	9527	(25.9%)	1.04 (0.87-1.23)	0.70	0.99 (0.82-1.18)	-0.157	0.88
6. Radiation exposure risk:							
No	31179	(84.7%)	1 (ref.)		1 (ref.)		
Yes	5628	(15.3%)	1.35 (1.11-1.64)	0.003	1.25 (1.03-1.53)	2.228	0.03
**Work conditions:**							
7. Deployment length:							
<1 month	16898	(45.9%)	1 (ref.)		1 (ref.)		
1-2 months	16126	(43.8%)	1.22 (1.03-1.45)	0.02	1.24 (1.04-1.49)	2.419	0.02
>= 3 months	3783	(10.3%)	1.85 (1.46-2.34)	< 0.001	1.52 (1.18-1.96)	3.256	0.001
8. Timing of leave:							
Early leave	22329	(60.7%)	1 (ref.)		1 (ref.)		
Late leave of work	12576	(34.2%)	1.25 (1.06-1.47)	0.009	1.10 (0.93-1.30)	1.075	0.28
No leave of work	1902	(5.2%)	1.81 (1.34-2.44)	< 0.001	1.33 (0.97-1.82)	1.761	0.08
9. Working overtime:							
Little to none	25420	(69.1%)	1 (ref.)		1 (ref.)		
Yes, < 3 months	8311	(22.6%)	1.48 (1.24-1.77)	< 0.001	1.35 (1.13-1.62)	3.228	0.001
Yes, >= 3 months	3076	(8.4%)	1.76 (1.39-2.25)	< 0.001	1.56 (1.22-2.01)	3.481	< 0.001

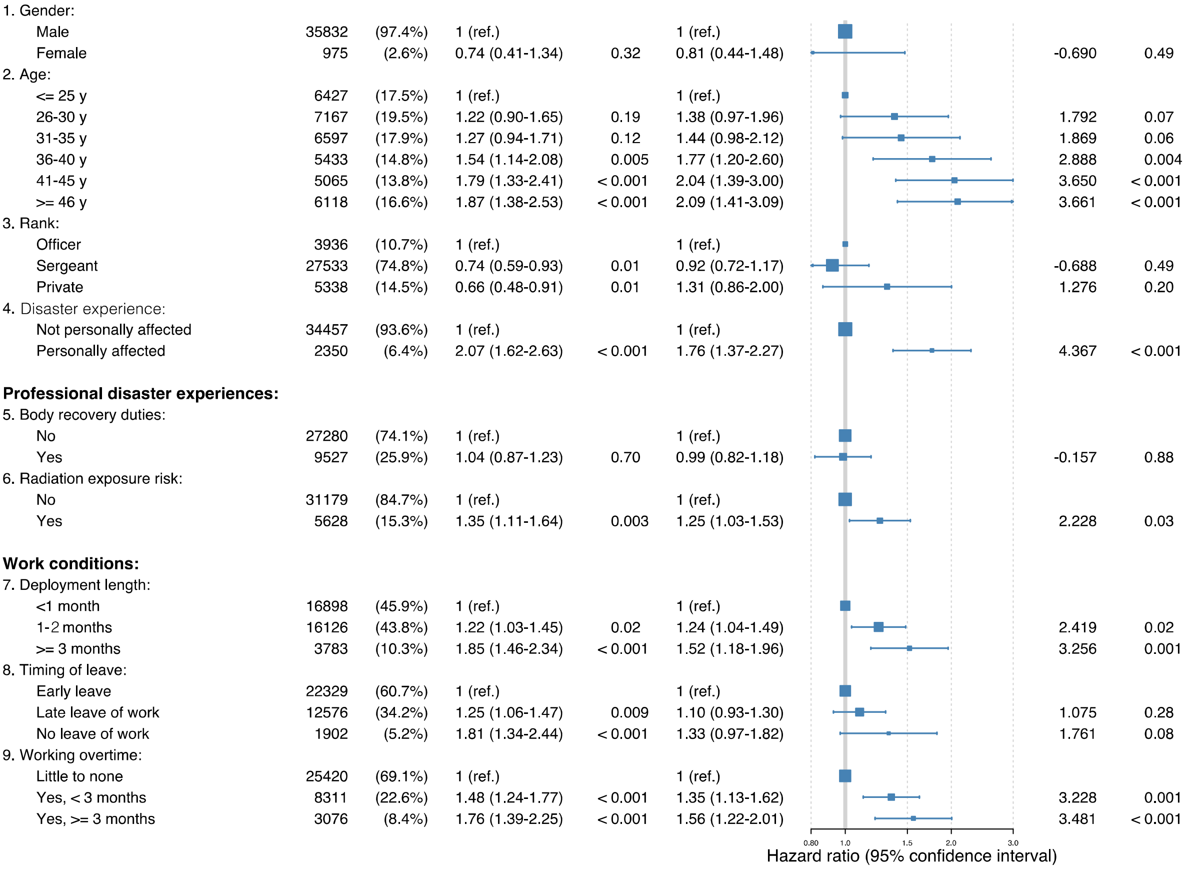

Forest plot of the results for those whose baseline IES-R score was 0 to 4. Adjusted hazard ratios (with 95% confidence intervals) and test statistics (*z* values) are shown for the 9 baseline predictors for incidence of probable PTSD using a multivariate Cox proportional-hazards model. It suggests that baseline IES-R is not confounding the relationships with high IES-R at follow up.

©2020 Nagamine M et al. *JAMA Network Open*.

eFigure 9. Sensitivity Analysis in Stratum With Baseline IES-R Scores >4

Independent variable	n	%	Crude HR	p-value	Adjusted HR	z-value	p-value
**Sociodemographics:**							
1. Gender:							
Male	14295	(97.0%)	1 (ref.)		1 (ref.)		
Female	445	(3.0%)	1.19 (0.85-1.68)	0.31	1.29 (0.91-1.82)	1.416	0.16
2. Age:							
<= 25 y	2774	(18.8%)	1 (ref.)		1 (ref.)		
26-30 y	2619	(17.8%)	1.20 (0.94-1.52)	0.14	1.35 (1.01-1.79)	2.056	0.04
31-35 y	2332	(15.8%)	1.50 (1.19-1.89)	0.001	1.69 (1.23-2.32)	3.243	0.001
36-40 y	2103	(14.3%)	1.82 (1.45-2.29)	< 0.001	2.07 (1.51-2.83)	4.513	< 0.001
41-45 y	2110	(14.3%)	1.86 (1.48-2.33)	< 0.001	2.12 (1.55-2.91)	4.680	< 0.001
>= 46 y	2802	(19.0%)	1.93 (1.54-2.41)	< 0.001	2.11 (1.54-2.89)	4.630	< 0.001
3. Rank:							
Officer	1682	(11.4%)	1 (ref.)		1 (ref.)		
Sergeant	10516	(71.3%)	0.74 (0.62-0.87)	< 0.001	0.91 (0.76-1.09)	-1.004	0.32
Private	2542	(17.2%)	0.58 (0.46-0.73)	< 0.001	1.31 (0.94-1.82)	1.608	0.11
4. Disaster experience:							
Not personally affected	12775	(86.7%)	1 (ref.)		1 (ref.)		
Personally affected	1965	(13.3%)	1.93 (1.67-2.23)	< 0.001	1.61 (1.38-1.89)	5.954	< 0.001
**Professional disaster experiences:**							
5. Body recovery duties:							
No	8968	(60.8%)	1 (ref.)		1 (ref.)		
Yes	5772	(39.2%)	1.04 (0.92-1.17)	0.56	1.04 (0.92-1.19)	0.623	0.53
6. Radiation exposure risk:							
No	12003	(81.4%)	1 (ref.)		1 (ref.)		
Yes	2737	(18.6%)	1.19 (1.03-1.38)	0.02	1.10 (0.95-1.28)	1.246	0.21
**Work conditions:**							
7. Deployment length:							
<1 month	4629	(31.4%)	1 (ref.)		1 (ref.)		
1-2 months	7153	(48.5%)	1.06 (0.92-1.23)	0.42	1.06 (0.91-1.24)	0.757	0.45
>= 3 months	2958	(20.1%)	1.68 (1.43-1.98)	< 0.001	1.32 (1.10-1.58)	2.990	0.003
8. Timing of leave:							
Early leave	7825	(53.1%)	1 (ref.)		1 (ref.)		
Late leave of work	5758	(39.1%)	1.33 (1.17-1.51)	< 0.001	1.15 (1.00-1.31)	2.030	0.04
No leave of work	1157	(7.8%)	1.98 (1.63-2.41)	< 0.001	1.41 (1.14-1.73)	3.201	0.001
9. Working overtime:							
Little to none	9301	(63.1%)	1 (ref.)		1 (ref.)		
Yes, < 3 months	3905	(26.5%)	1.38 (1.21-1.58)	< 0.001	1.26 (1.09-1.45)	3.212	0.001
Yes, >= 3 months	1534	(10.4%)	1.67 (1.40-1.99)	< 0.001	1.43 (1.19-1.73)	3.799	< 0.001

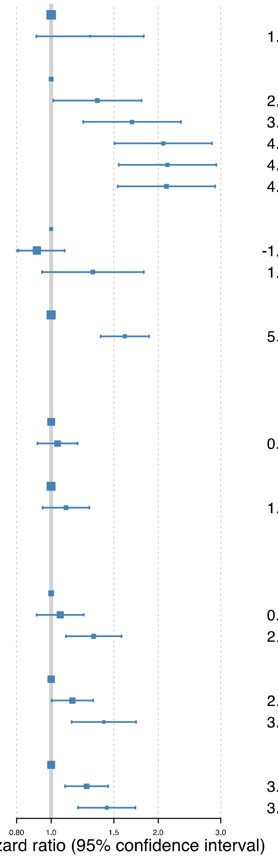

Forest plot of the results for those whose baseline IES-R score was 5 to 25. Adjusted hazard ratios (with 95% confidence intervals) and test statistics (*z* values) are shown for the 9 baseline predictors for incidence of probable PTSD using a multivariate Cox proportional-hazards model. It suggests that baseline IES-R is not confounding the relationships with high IES-R at follow up.

©2020 Nagamine M et al. *JAMA Network Open*.

eFigure 10. Sensitivity Analysis in Stratum of Those Not Being Personally Affected by the Disaster

Independent variable	n	%	Crude HR	p-value	Adjusted HR	z-value	p-value
**Sociodemographics:**							
1. Gender:							
Male	45988	(97.4%)	1 (ref.)		1 (ref.)		
Female	1244	(2.6%)	1.01 (0.72-1.43)	0.93	1.23 (0.86-1.74)	1.137	0.26
2. Age:							
<= 25 y	8561	(18.1%)	1 (ref.)		1 (ref.)		
26-30 y	8968	(19.0%)	1.10 (0.90-1.35)	0.36	1.31 (1.02-1.67)	2.158	0.03
31-35 y	8100	(17.1%)	1.25 (1.03-1.53)	0.03	1.52 (1.16-1.99)	3.077	0.002
36-40 y	6883	(14.6%)	1.59 (1.31-1.94)	< 0.001	1.93 (1.48-2.52)	4.858	< 0.001
41-45 y	6574	(13.9%)	1.75 (1.44-2.13)	< 0.001	2.11 (1.62-2.75)	5.514	< 0.001
>= 46 y	8146	(17.2%)	1.81 (1.49-2.21)	< 0.001	2.09 (1.60-2.74)	5.371	< 0.001
3. Rank:							
Officer	4990	(10.6%)	1 (ref.)		1 (ref.)		
Sergeant	34943	(74.0%)	0.73 (0.63-0.86)	< 0.001	0.92 (0.78-1.08)	-0.992	0.32
Private	7299	(15.5%)	0.65 (0.53-0.81)	< 0.001	1.38 (1.03-1.84)	2.193	0.03
4. Disaster experience:							
Not personally affected	47232	(100.0%)					
Personally affected							
**Professional disaster experiences:**							
5. Body recovery duties:							
No	33439	(70.8%)	1 (ref.)		1 (ref.)		
Yes	13793	(29.2%)	1.30 (1.16-1.45)	< 0.001	1.23 (1.10-1.38)	3.510	< 0.001
6. Radiation exposure risk:							
No	39984	(84.7%)	1 (ref.)		1 (ref.)		
Yes	7248	(15.3%)	1.32 (1.16-1.51)	< 0.001	1.22 (1.07-1.40)	2.906	0.004
**Work conditions:**							
7. Deployment length:							
<1 month	20355	(43.1%)	1 (ref.)		1 (ref.)		
1-2 months	21890	(46.3%)	1.29 (1.14-1.45)	< 0.001	1.26 (1.11-1.42)	3.624	< 0.001
>= 3 months	4987	(10.6%)	2.29 (1.96-2.67)	< 0.001	1.93 (1.64-2.27)	7.958	< 0.001
8. Timing of leave:							
Early leave	28577	(60.5%)	1 (ref.)		1 (ref.)		
Late leave of work	16175	(34.2%)	1.29 (1.15-1.44)	< 0.001	1.14 (1.02-1.28)	2.254	0.02
No leave of work	2480	(5.3%)	2.28 (1.89-2.75)	< 0.001	1.70 (1.40-2.06)	5.299	< 0.001
9. Working overtime:							
Little to none	31943	(67.6%)	1 (ref.)		1 (ref.)		
Yes, < 3 months	11115	(23.5%)	1.55 (1.37-1.74)	< 0.001	1.41 (1.24-1.59)	5.444	< 0.001
Yes, >= 3 months	4174	(8.8%)	1.90 (1.62-2.23)	< 0.001	1.64 (1.39-1.94)	5.875	< 0.001

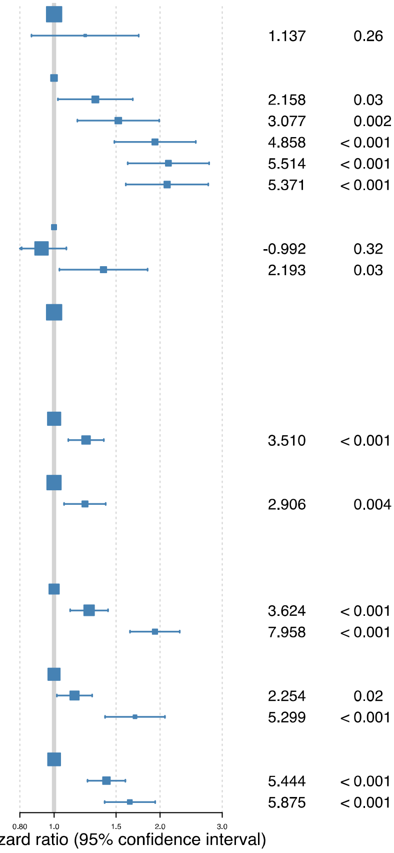

Forest plot of the results for those 47232 not being personally affected by the disaster. Adjusted hazard ratios (with 95% confidence intervals) and test statistics (*z* values) are shown for the 9 baseline predictors for incidence of probable PTSD using a multivariate Cox proportional-hazards model.

©2020 Nagamine M et al. *JAMA Network Open*.

eFigure 11. Sensitivity Analysis in Stratum of Those Being Personally Affected by the Disaster

Independent variable	n	%	Crude HR	p-value	Adjusted HR	z-value	p-value
**Sociodemographics:**							
1. Gender:							
Male	4139	(95.9%)	1 (ref.)		1 (ref.)		
Female	176	(4.1%)	1.04 (0.58-1.84)	0.91	1.18 (0.66-2.12)	0.552	0.58
2. Age:							
<= 25 y	640	(14.8%)	1 (ref.)		1 (ref.)		
26-30 y	818	(19.0%)	1.32 (0.82-2.12)	0.26	1.65 (0.97-2.81)	1.830	0.07
31-35 y	829	(19.2%)	1.44 (0.90-2.29)	0.13	2.01 (1.10-3.65)	2.284	0.02
36-40 y	653	(15.1%)	1.81 (1.14-2.90)	0.01	2.49 (1.36-4.54)	2.962	0.003
41-45 y	601	(13.9%)	2.01 (1.26-3.21)	0.003	2.80 (1.53-5.12)	3.334	0.001
>= 46 y	774	(17.9%)	2.46 (1.56-3.87)	< 0.001	3.34 (1.83-6.09)	3.937	< 0.001
3. Rank:							
Officer	628	(14.6%)	1 (ref.)		1 (ref.)		
Sergeant	3106	(72.0%)	0.77 (0.57-1.03)	0.08	0.97 (0.71-1.32)	-0.199	0.84
Private	581	(13.5%)	0.72 (0.46-1.10)	0.13	1.83 (1.02-3.27)	2.026	0.04
4. Disaster experience:							
Not personally affected							
Personally affected	4315	(100.0%)					
**Professional disaster experiences:**							
5. Body recovery duties:							
No	2809	(65.1%)	1 (ref.)		1 (ref.)		
Yes	1506	(34.9%)	1.08 (0.85-1.36)	0.54	1.05 (0.82-1.33)	0.375	0.71
6. Radiation exposure risk:							
No	3198	(74.1%)	1 (ref.)		1 (ref.)		
Yes	1117	(25.9%)	1.03 (0.80-1.33)	0.81	1.04 (0.81-1.35)	0.324	0.75
**Work conditions:**							
7. Deployment length:							
<1 month	1172	(27.2%)	1 (ref.)		1 (ref.)		
1-2 months	1389	(32.2%)	1.45 (1.06-1.99)	0.02	1.44 (1.04-1.99)	2.213	0.03
>= 3 months	1754	(40.6%)	1.56 (1.15-2.11)	0.004	1.44 (1.05-1.98)	2.264	0.02
8. Timing of leave:							
Early leave	1577	(36.5%)	1 (ref.)		1 (ref.)		
Late leave of work	2159	(50.0%)	1.38 (1.07-1.78)	0.01	1.26 (0.98-1.63)	1.773	0.08
No leave of work	579	(13.4%)	1.42 (0.99-2.02)	0.05	1.12 (0.78-1.63)	0.625	0.53
9. Working overtime:							
Little to none	2778	(64.4%)	1 (ref.)		1 (ref.)		
Yes, < 3 months	1101	(25.5%)	1.45 (1.12-1.86)	0.004	1.29 (1.00-1.67)	1.932	0.05
Yes, >= 3 months	436	(10.1%)	1.70 (1.22-2.38)	0.002	1.53 (1.08-2.17)	2.417	0.02

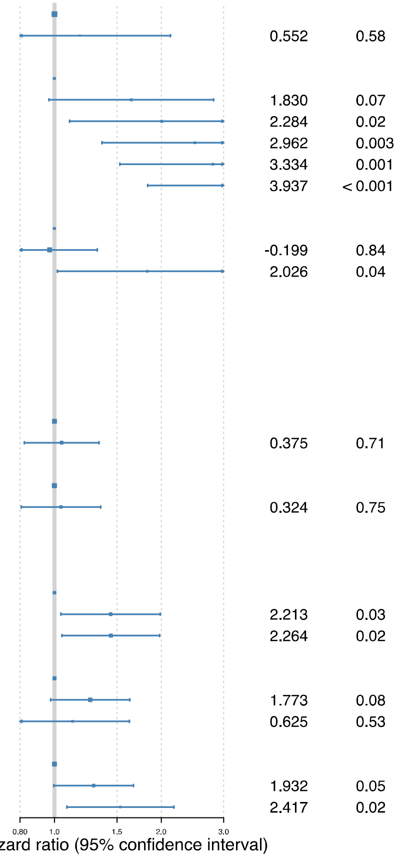

Forest plot of the results for those 4315 being personally affected by the disaster. Adjusted hazard ratios (with 95% confidence intervals) and test statistics (*z* values) are shown for the 9 baseline predictors for incidence of probable PTSD using a multivariate Cox proportional-hazards model.

©2020 Nagamine M et al. *JAMA Network Open*.

eTable 1. Comparison of the Initial Survey Data for Participants With and Without Follow-up After the 2-Year Survey Point

	Participants who dropped out after 2-year survey point	Participants followed after 2-year survey point at least once	Total Participants	*P* value
**Total number (%)**	5408 (9.6)	50 980 (90.4)	56 388 (100.0)	
**Survey at 1-month survey point**				
No. (%)	5053 (9.4)	48 647 (90.6)	53 700 (100.0)	
IES-R, mean (SD)	5.3 (8.1)	4.5 (7.2)	4.6 (7.3)	<.001
Probable PTSD (IES-R ≥25), No. (%)	188 (3.7)	1287 (2.6)	1475 (2.7)	<.001
**Survey at 6-month survey point**				
No. (%)	5290 (9.6)	49 865 (90.4)	55 155 (100.0)	
IES-R, mean (SD)	4.2 (7.0)	3.5 (6.1)	3.6 (6.2)	<.001
Probable PTSD (IES-R ≥25), No. (%)	122 (2.3)	819 (1.6)	941 (1.7)	<.001
**Survey at 12-month survey point**				
No. (%)	5310 (9.5)	50 696 (90.5)	56 006 (100.0)	
IES-R, mean (SD)	3.3 (5.9)	2.8 (5.4)	2.9 (5.4)	<.001
Probable PTSD (IES-R ≥25), No. (%)	74 (1.4)	570 (1.1)	644 (1.1)	0.080
**Gender, No. (%)**				<.001
Male	5096 (94.2)	49 672 (97.4)	54 768 (97.1)	
Female	312 (5.8)	1308 (2.6)	1620 (2.9)	
**Age at baseline, No. (%), y**				<.001
≤25	2010 (37.2)	8199 (16.1)	10 209 (18.1)	
26-30	651 (12.0)	9908 (19.5)	10 559 (18.7)	
31-35	242 (4.5)	9395 (18.4)	9637 (17.1)	
36-40	226 (4.2)	8005 (15.7)	8231 (14.6)	
41-45	218 (4.0)	7655 (15.0)	7873 (14.0)	
≥46	2057 (38.1)	7774 (15.3)	9831 (17.4)	
**Rank, No. (%)**				<.001
Officer	710 (13.1)	5688 (11.2)	6398 (11.3)	
Sergeant	2412 (44.6)	38 793 (76.1)	41 205 (73.1)	
Private	2286 (42.3)	6499 (12.7)	8785 (15.6)	
**Personal experience of the disaster, No. (%)**				.393
No	4910 (90.8)	46 446 (91.2)	51 356 (91.2)	
Yes	495 (9.2)	4488 (8.8)	4983 (8.8)	
**Body recovery duties, No. (%)**				<.001
No	4073 (75.3)	35 562 (69.8)	39 635 (70.3)	
Yes	1335 (24.7)	15 399 (30.2)	16 734 (29.7)	
**Radiation exposure risk, No. (%)**				<.001
No	4622 (85.5)	42 431 (83.3)	47 053 (83.5)	
Yes	785 (14.5)	8518 (16.7)	9303 (16.5)	
**Deployment length, No. (%), month**				<.001
<1	2580 (47.9)	21 029 (41.3)	23 609 (41.9)	
1-2	2030 (37.7)	22 972 (45.1)	25 002 (44.4)	
≥3	780 (14.5)	6966 (13.7)	7746 (13.7)	
**Post-deployment leave, No. (%)**				<.001
Within 2 weeks	2771 (52.5)	29 458 (58.2)	32 229 (57.7)	
Over 2 weeks	2067 (39.2)	18 090 (35.8)	20 157 (36.1)	
No leave taken	441 (8.4)	3024 (6.0)	3465 (6.2)	
**Post-deployment overtime work, No. (%)**				<.001
Little to none	3773 (71.5)	33 590 (66.5)	37 363 (66.9)	
Yes, overwork <3 months	1133 (21.5)	12 187 (24.1)	13 320 (23.9)	
Yes, overwork ≥3 months	373 (7.1)	4763 (9.4)	5136 (9.2)	

©2020 Nagamine M et al. *JAMA Network Open*.

eTable 2. Trends in the Type of Probable PTSD at Baseline (n = 1475) Classified by the Way of Changes in Their PTSD Symptoms

	6 months	12 months	24 months	36 months	48 months	60 months	72 months
Participants followed	1426 (96.7)	1465 (99.3)	1060 (71.9)	805 (54.6)	679 (46.0)	679 (46.0)	536 (36.3)
Participants recovered	1053 (73.8)	1197 (81.7)	907 (85.6)	724 (89.9)	621 (91.5)	597 (87.9)	491 (91.6)
Participants persisted	373 (26.2)	183 (12.5)	74 (7.0)	47 (5.8)	28 (4.1)	38 (5.6)	20 (3.7)
Participants recurrent	—	85 (5.8)	79 (7.5)	34 (4.2)	30 (4.4)	44 (6.5)	25 (4.7)

Note: Participants were classified into "Recovered" if they scored <25 on IES-R, "Persisted" if they scored ≥25 on IES-R continuously, and "Recurrent" if they scored ≥25 on IES-R, but scored <25 on immediately before the IES-R.

©2020 Nagamine M et al. *JAMA Network Open*.
